# Tandemly Repeated G-Quadruplex Structures in the Pseudorabies Virus Genome: Implications for Epiberberine-Based Antiviral Therapy

**DOI:** 10.3390/ijms26083764

**Published:** 2025-04-16

**Authors:** Songjie Fan, Xiaotian Chang, Yan Qiao, Xiaoxiao Zhao, Jiafu Zhao, Heshui Zhu, Yingqian Han, Chao Zhang

**Affiliations:** College of Veterinary Medicine, Henan Agricultural University, Zhengzhou 450046, China; 13525724290@163.com (S.F.); 13938606732@163.com (X.C.); 18860361915@163.com (Y.Q.); zxx15039939436@163.com (X.Z.); 19503837332@163.com (J.Z.); zhuheshui@126.com (H.Z.); twgjl@163.com (Y.H.)

**Keywords:** DNAG-quadruplex, pseudorabies virus, epiberberine, antiviral activity

## Abstract

G-quadruplex (G4) structures have emerged as critical regulatory elements in viral genomes and represent potential targets for antiviral intervention. In this study, we identified and characterized G4 structures in the unique long (UL) region of the Pseudorabies virus (PRV) genome, highlighting their role as novel antiviral targets. Bioinformatic analysis revealed two guanine-rich regions (R1 and R2) that form stable G4 structures, as confirmed by fluorescence assays, circular dichroism (CD) spectroscopy, and immunofluorescence staining. Notably, these G4 structures exhibit a tandem repeat arrangement, a previously unreported feature in the PRV genome. Epiberberine (EPI), a natural G4-stabilizing ligand, bound to and stabilized these structures, leading to the inhibition of Taq polymerase progression. Functional assays demonstrated that EPI effectively suppressed PRV replication in vitro while having no significant impact on viral entry or release. In vivo, EPI treatment significantly improved survival rates and reduced viral loads in multiple organs, including the brain, heart, lungs, and kidneys of infected mice. These findings provide new insights into the role of G4 structures in PRV replication and demonstrate that EPI exhibits potential antiviral activity by targeting G4 structures.

## 1. Introduction

Guanine-rich DNA or RNA can fold into a noncanonical secondary structure known as a G4 structure. G4 DNA is composed of stacked G-quartets, which are formed by the interaction of four guanine bases through Hoogsteen bonds. These structures are stabilized by some metal ions, such as potassium (K), calcium (Ca), magnesium (Mg), sodium (Na), and barium (Ba) [1,2]. G4 structures play crucial biological roles, including the regulation of essential processes, such as DNA replication, transcription, and translation [3,4,5]. Increasing evidence indicates that G4 DNA is widely distributed across the genomes of mammals, plants, fungi, and bacteria [6,7,8,9]. Moreover, a growing number of studies have reported the presence of G4-forming sequences in viral genomes, including those of human immunodeficiency virus (HIV) [10], Epstein–Barr virus (EBV) [11], human papillomavirus (HPV) [12], human cytomegalovirus (HCMV) [13], herpes simplex virus (HSV) [14], Nipah virus [15], hepatitis C virus (HCV) [16], Zika virus [17], Hepatitis B virus (HBV) [18,19,20], Monkeypox virus (MPXV) [21], Kaposi’s sarcoma-associated herpes virus(KSHV) [22], SARS-CoV [23], African swine fever virus (ASFV) [24,25], influenza A virus [26,27], enterovirus [28], and SARS-CoV-2 [29,30]. The formation of G4 structures within these viral genomes can influence key viral processes, such as genome replication, transcription, and translation.

Pseudorabies virus (PRV), a member of the Alphaherpesvirinae subfamily, is the causative agent of Aujeszky’s disease, which has led to significant economic losses in the global swine industry [31,32]. In addition to infecting pigs, PRV has the ability to infect a wide range of other animals, including cats, rabbits, dogs, cattle, sheep, goats, lynxes, foxes, and minks [33]. The Bartha-K61 vaccine, which was introduced from Hungary, has been effective in controlling the PRV epidemic in China. However, since 2011, highly pathogenic variants of PRV have emerged in China, against which the traditional Bartha-K61 vaccine does not offer complete protection [34,35]. Importantly, recent clinical reports have raised concerns about the zoonotic potential of PRV [36]. Specifically, PRV strain hSD-1/2019 was isolated from the cerebrospinal fluid of a human patient [37], providing direct evidence of PRV transmission to humans. This finding underscores PRV’s emerging role as a public health pathogen. Additionally, it is important to note that PRV can establish latent infections in the peripheral ganglia of adult pigs [38], making it difficult to eradicate. Given these challenges, there is an urgent need to develop new control strategies and antiviral drugs to combat PRV infections.

Previous research by our group and others has identified G4-forming sequences within the PRV genome, and it has been demonstrated that small molecule compounds targeting these structures can inhibit viral proliferation [39,40,41]. In addition to the high guanine content characteristic of the PRV genome, another notable feature is the presence of multiple repetitive sequence regions. These repetitive regions are typically located between genes, and their specific functions remain largely unexplored. Studies on viruses homologous to PRV, such as herpes simplex virus (HSV), have reported that the repetitive sequence regions in their genomes can form G4 structures. For example, the small molecule compound BRACO-19 has been shown to recognize and bind to these G4 structures, subsequently affecting viral replication within cells [14]. Furthermore, research on the varicella-zoster virus (VZV) has revealed that the G-rich repetitive sequences within the gC gene can form G4 structures, which in turn inhibit gC gene expression and reduce the spread of the virus between cells [42].

Epiberberine, a natural protoberberine alkaloid extracted from the traditional Chinese medicinal herb *Coptis chinensis Franch*, has demonstrated potential in treating a variety of chronic diseases, including antidiabetic, antihyperlipidemic, and anti-inflammatory effects [43]. Notably, studies have reported that berberine, an isomer of epiberberine, can specifically recognize and bind to G4 structures, inhibiting tumor growth and positioning it as a potential antitumor agent in traditional Chinese medicine [44,45,46]. Moreover, EPI itself has been shown to recognize and bind to hybrid G4 structures formed by telomericDNA [47,48]. However, the potential biological functions of EPI, through its binding to G4 structures, have not been explored.

In this study, we identified two conserved G4-forming sequences within the repetitive regions of the PRV DNA genome. Through a series of biophysical and biochemical experiments, we demonstrated that these guanine-rich sequences are capable of folding into hybrid-type G4 structures. Notably, these G4 structures can be bound and stabilized by EPI. When EPI was introduced into PRV-infected cells, we observed a significant inhibition of virus DNA replication, suggesting its potential antiviral activity. Furthermore, in vivo experiments provided additional evidence that EPI can effectively suppress PRV infection. These findings collectively suggest that the G4 structures within the PRV genome may serve as promising targets for the development of novel therapeutic strategies against PRV.

## 2. Results

### 2.1. Identification of Tandemly Arranged G-Quadruplex Sequences in the PRV Genome

**Figure 1:**
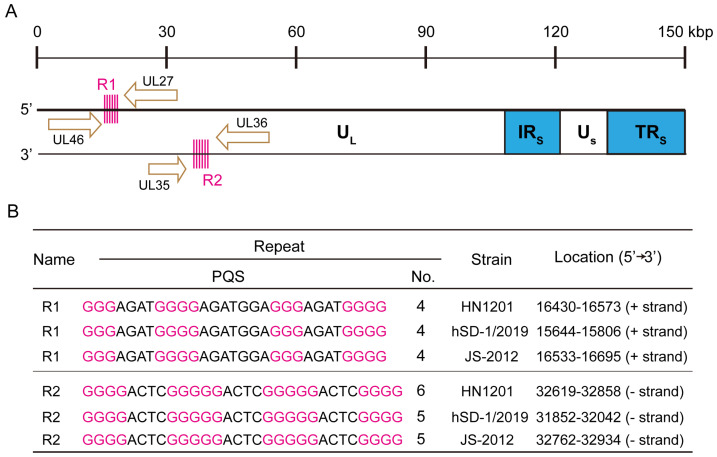
Identification of multiple G4-forming sequences in the PRV genome. (**A**) Schematic representation of the PRV genome. The arrow indicates the transcription direction. (**B**) The genomic sequences of three representative PRV variants, HN1201(KP722022.1), HSD-1/2019 (MT468550.1), and JS-2012 (KP257591.1), were retrieved from NCBI. The potential G-quadruplex-forming sequences (PQS) were analyzed using the QGRS Mapper tool, and the guanine bases involved in the formation of the G-quadruplex are denoted in pink.

The PRV genome is characterized by a high GC content (68–74%) and contains various repetitive DNA sequences [49]. We aimed to investigate the distribution of putative quadruplex sequences (PQS) within the PRV genome. To predict G4 formation, we employed the QGRS Mapper online tool [50] (https://bioinformatics.ramapo.edu/QGRS/index.php, accessed on 15 December 2023), setting the guanine repeat number to 3 and varying the loop length from 1 to 11 nucleotides. Notably, we identified two repeat regions in the UL region of the PRV genome, composed of G4-forming sequences arranged in a tandem manner. One of these repeated G4 regions, located on the sense strand (+), consisted of the G4 sequence 5′-GGGGAGAGGGGAGACGAGAGGGGAGAGGGG, which we designated as R1-PQS. The R1-PQS repeat region is located between the UL46 and UL27 genes and is situated on the template strand for UL27 transcription. In addition, for the HN1201 strain, this repeat region consists of four repeated PQS sequences. The other repeated G4 region, located on the antisense strand (−), consisted of the G4 sequence 5′-GGGGACTCGGGGGACTCGGGGGACTCGGGG, which we termed R2-PQS. The R2-PQS repeat region is located between the UL35 and UL36 genes and is situated on the template strand for UL35 transcription. For the HN1201 strain, this repeat region consists of six repeated PQS sequences. Furthermore, these two repeat regions were highly conserved across three representative PRV variants: HN1201, JS-2012, and hSD-1/2019 (Figure 1A,B). The sequences of the repeated G4s are provided in Table S1, highlighted in yellow.

### 2.2. R1-PQS and R2-PQS Repeats Fold into Hybrid G-Quadruplex Structures

**Figure 2:**
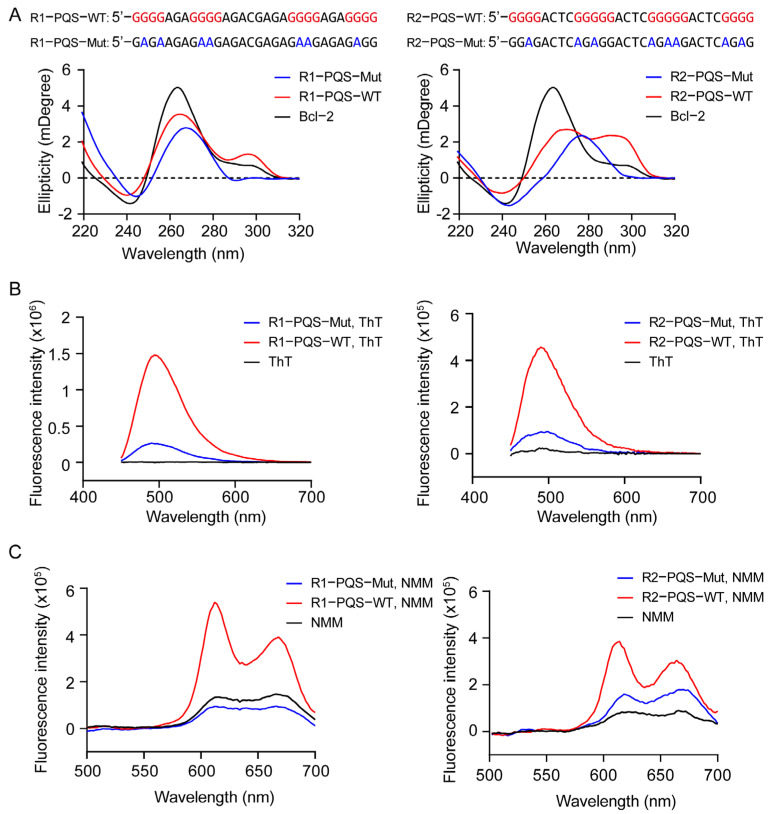
Characterization of G4 formation in R1-PQS and R2-PQS regions. (**A**) Wild-type (WT) and mutant (Mut) oligonucleotides corresponding to the R1-PQS and R2-PQS regions were analyzed by CD spectroscopy. (**B**) WT and Mut oligonucleotides were incubated with Thioflavin T (THT), and fluorescence was measured to assess G4 formation. (**C**) WT and Mut oligonucleotides were incubated with N-methylmesoporphyrin IX (NMM), and fluorescence was recorded to confirm G4 structure formation.

The CD spectroscopy has been widely used to identify G4 formation and determine G4 conformations [51]. R1-PQS and R2-PQS were annealed in a buffer containing 50 mM K+ and subjected to CD analysis. Both oligonucleotides displayed a negative peak at 240 nm, a positive peak at 264 nm, and another positive peak at 294 nm, which are characteristic of a hybrid G4 structure. These spectral features were similar to those observed in the well-studied hybrid G4 structure of Bcl-2 [52]. However, when the core guanine residues were mutated to adenine to disrupt G4 formation, the CD signature was lost in both the negative controls, R1-PQS-Mut and R2-PQS-Mut (Figure 2A). To further corroborate G4 formation in R1-PQS and R2-PQS, a fluorescence turn-on assay was performed using the fluorescent probes ThT and NMM, which enhance fluorescence emission upon binding to G4 structures [53,54,55]. As expected, compared to the negative controls, both ThT and NMM showed significantly increased fluorescence intensity when incubated with annealed R1-PQS and R2-PQS (Figure 2B,C).

### 2.3. EPI Binds to and Stabilizes G4 Structures Formed in R1-PQS and R2-PQS

**Figure 3:**
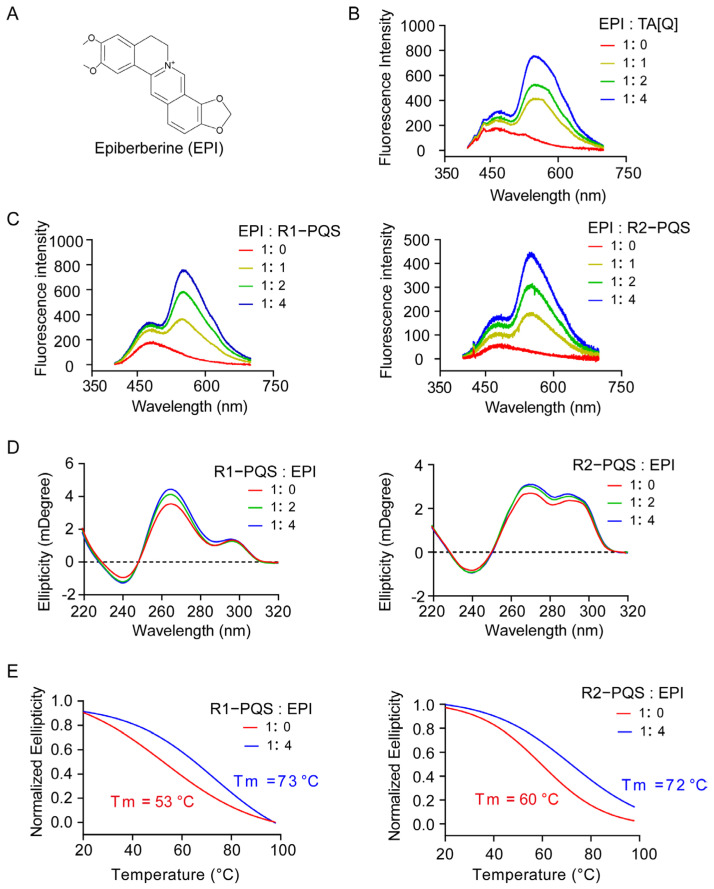
Epiberberine binding and stabilization of G-quadruplexes formed by R1-PQS and R2-PQS. (**A**) Molecular structure of EPI. (**B**,**C**) The telomeric sequence (TA[Q]) was used as a positive control. G4 formation was assessed by fluorescence after incubation of R1-PQS, R2-PQS, and TA[Q] with EPI. (**D**) The structural changes of R1-PQS and R2-PQS upon EPI binding were analyzed by CD spectroscopy. (**E**) The thermal stability of R1-PQS and R2-PQS with EPI binding was evaluated by CD melting curves, with the melting temperature (Tm) determined by fitting the data to a sigmoidal curve using the Boltzmann function.

A previous study showed that EPI can bind the hybrid G4 formed by the human telomere sequence TA[Q] [56]. We aimed to investigate whether EPI can similarly recognize and bind the hybrid G4 structures formed in R1-PQS and R2-PQS. First, a fluorescence assay was conducted to assess this interaction. Consistent with the positive control, where EPI enhanced fluorescence emission upon binding to the G4 formed by TA[Q], EPI exhibited a significant increase in fluorescence when incubated with both annealed R1-PQS and R2-PQS (Figure 3A–C). Moreover, EPI did not alter the CD spectral characteristics but increased the peak at 264 nm, suggesting that EPI can enhance the thermal stability of the G4 structures (Figure 3D). To further validate this observation, we conducted a CD melting assay. When 1 equivalent and 2 equivalents of EPI were added to the samples, the melting temperature (Tm) increased from 53 °C to 73 °C for R1-PQS and from 60 °C to 72 °C for R2-PQS, respectively (Figure 3E).

These results collectively demonstrate that EPI can bind and stabilize the G4 structures formed in R1-PQS and R2-PQS.

### 2.4. R1-PQS and R2-PQS Form G4 Structures in Cells

**Figure 4:**
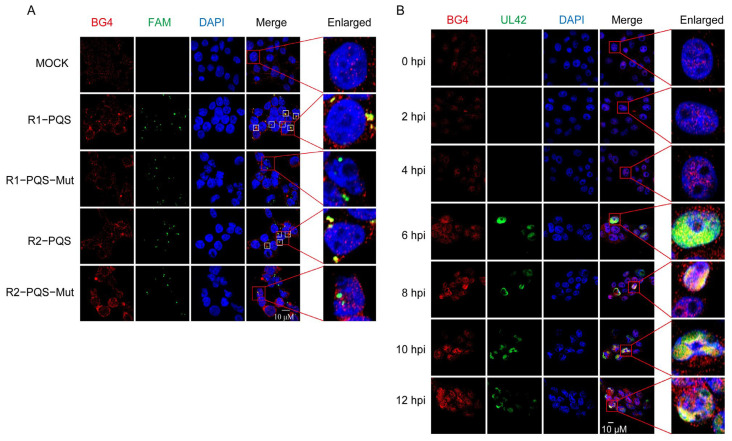
R1-PQS and R2-PQS form G-quadruplexes in cells. (**A**) FAM-labeled R1-PQS, R1-PQS-Mut, R2-PQS, and R2-PQS-Mut oligonucleotides were transfected into 293T cells and stained with BG4 antibody (red) to detect G4 structures. Co-localization of G4 structures and BG4 is shown in yellow. White boxes indicate regions of co-localization, while red boxes highlight areas shown at higher magnification in the insets. (**B**) PK-15 cells infected with PRV-HN1201 were stained with BG4 antibody (red) to detect G4 structures and anti-UL42 antibody (green) to detect the UL42 protein. DAPI (blue) was used to stain the cell nuclei. Red boxes indicate regions that are enlarged in the insets. Scale bar: 10 µm. Images were captured by fluorescence microscopy.

To verify that R1-PQS and R2-PQS can form G4 structures in cells, we transfected FAM-labeled R1-PQS and R2-PQS, along with their mutated counterparts (R1-PQS-Mut and R2-PQS-Mut) into 293T cells. G4 structures were then detected using the widely recognized G4-binding antibody BG4 [57]. Compared to the mutated versions (R1-PQS-Mut and R2-PQS-Mut), the BG4 antibody (red fluorescence) co-localized with FAM-labeled wild-type R1-PQS and R2-PQS (green fluorescence), resulting in a prominent yellow fluorescence signal (Figure 4A). This co-localization indicates that R1-PQS and R2-PQS oligonucleotides form G4 structures within the cells.

To further confirm that the PRV genome forms G4 structures in cells, we infected PK-15 cells with PRV-HN1201. The PRV UL42 protein, a DNA polymerase processivity factor essential for viral replication [58], was used as an indicator of viral genome replication. As shown in Figure 4B, the expression of UL42 increased over time, particularly at 6, 8, 10, and 12 h post-infection (hpi), and localized to the cell nucleus. This result suggests that the PRV genome undergoes replication within the nucleus during these time points. Notably, significant co-localization of BG4 and UL42 (yellow fluorescence signal) was observed, supporting the conclusion that G4 structures are formed during the replication of the PRV genome.

### 2.5. EPI Impairs DNA Synthesis by Targeting G4 Structures in R1-PQS and R2-PQS

**Figure 5:**
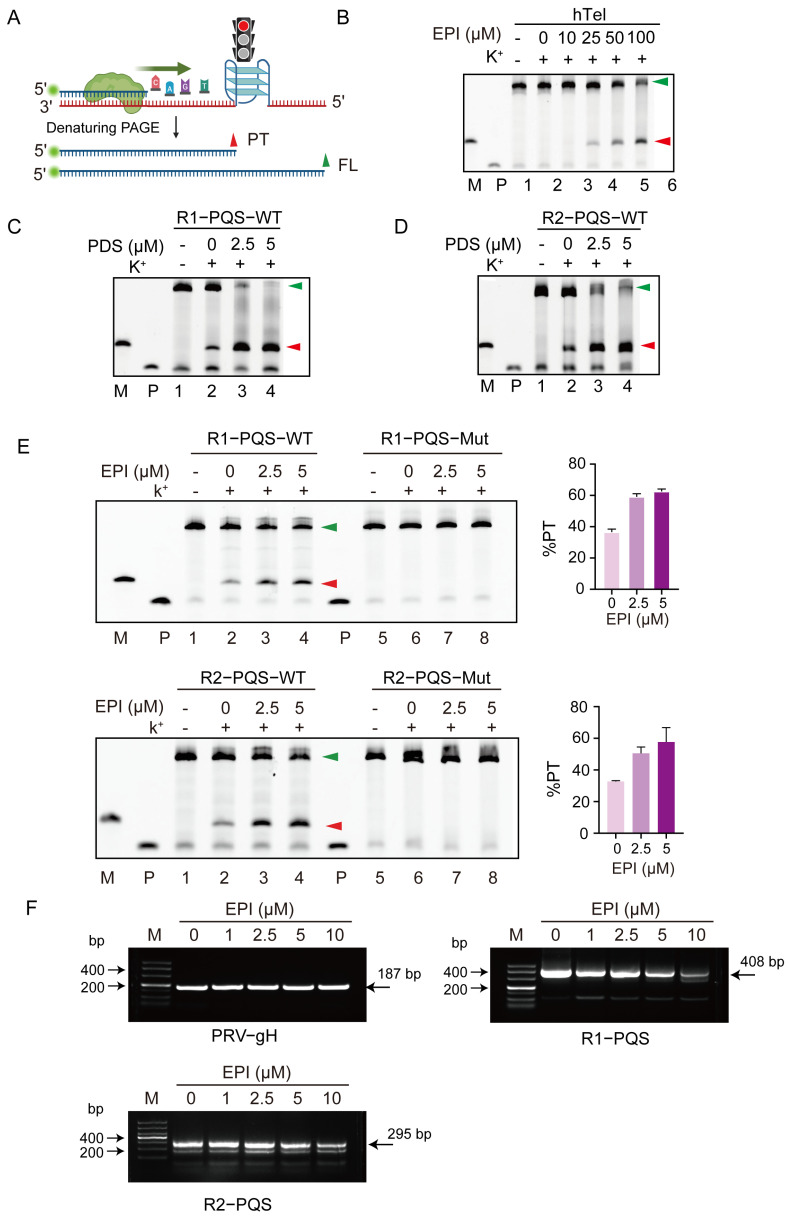
EPI inhibits DNA replication by targeting G4 structures formed by R1-PQS and R2-PQS. (**A**) Schematic of the Taq polymerase stop assay showing premature termination products (red arrows) due to G4-induced steric hindrance. Green arrows indicate the full-length products synthesized by Taq polymerase. (**B**) Primer extension with telomeric sequence (positive control) and varying concentrations of EPI to assess its effect on DNA replication. (**C**,**D**) Positive control using PDS, a G4 stabilizer, in the Taq polymerase stop assay. (**E**) Primer extension assays on wild-type and mutant R1-PQS and R2-PQS sequences with varying EPI concentrations. (**F**) PCR amplification of the PRV-HN1201 genome to assess G4 formation in the R1-PQS and R2-PQS regions upon EPI treatment. In (**B**,**E**), “−” indicates the absence of both K⁺ and EPI. In (**C**,**D**), “−” indicates the absence of both K⁺ and PDS. “+” indicates the presence of K⁺.

Next, we aimed to investigate the biological functions of the G4 structures formed within the R1-PQS and R2-PQS regions. To this end, we performed a Taq polymerase stop assay, which mimics the DNA replication process. Given the spatial hindrance effect of G4 structures, we anticipated that Taq polymerase would stall at the G4 sites, resulting in the accumulation of premature termination products alongside the full-length extension product (Figure 5A).

First, we used the telomere sequence as a positive control to validate the Taq polymerase stop assay. In this experiment, the addition of EPI substantially blocked the ability of Taq polymerase to bypass the G4 structure formed by the telomere sequence. This led to the production of premature termination products in addition to the full-length products (Figure 5B). This result is consistent with a previous study [59]. Subsequently, since pyridostatin (PDS) is a well-studied G4 stabilizer [60], we used PDS as a positive control ligand to investigate the impact of G4 structures formed at the R1-PQS and R2-PQS regions on Taq polymerase activity. As shown in Figure 5C,D, the addition of PDS significantly increased the production of premature termination products, confirming the inhibitory effect of G4 structures on Taq polymerase progression.

Next, we examined the effect of EPI on the G4 structures formed by the R1-PQS and R2-PQS regions. Notably, when varying concentrations of EPI were added to the reaction mix, the appearance of premature termination products increased in a concentration-dependent manner, suggesting that EPI effectively stabilizes G4 structures and impairs DNA synthesis. In contrast, no premature termination products were detected for the mutant versions of R1-PQS and R2-PQS, even in the presence of K+ and EPI (Figure 5E). This strongly indicates that EPI specifically binds to the G4 structures and disrupts DNA replication.

To further verify whether G4 structures are also formed in the PRV genome, we extracted PRV DNA from infected cells and incubated it with different concentrations of EPI in the presence of 50 mM K+. The mixture was annealed and then subjected to PCR. For both R1-PQS and R2-PQS, the PCR products, amplified using primers targeting the G4 motifs, decreased in an EPI concentration-dependent manner. As a control, the PCR product for the gH gene, which does not contain a G4 sequence, was unaffected by EPI (Figure 5F).

These results demonstrate that EPI inhibits DNA synthesis by specifically targeting the G4 structures formed within R1-PQS and R2-PQS.

### 2.6. Inhibition of PRV Proliferation by EPI in Infected Cells

**Figure 6:**
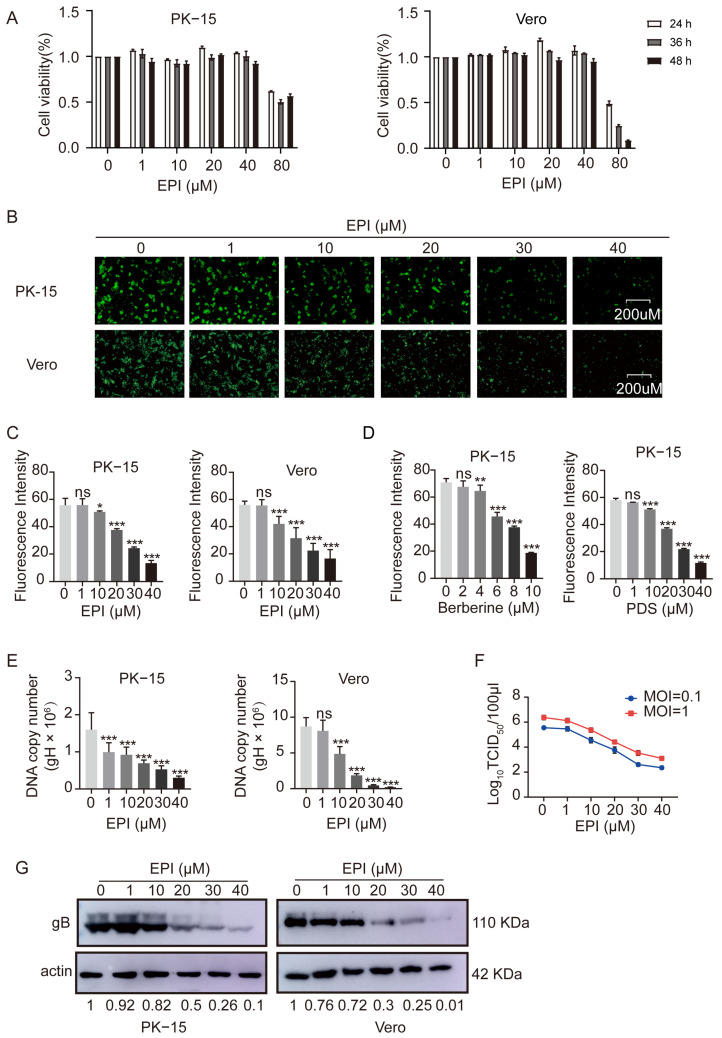
EPI inhibits PRV proliferation in cells. (**A**) PK-15 and Vero cells were treated with varying concentrations of EPI for 24, 36, or 48 h, and cell viability was measured using the CCK-8 assay. (**B**) PK-15 and Vero cells were pre-treated with different concentrations of EPI, followed by infection with PRV-GFP (MOI = 0.1). The GFP-positive cells were visualized using a fluorescence microscope. (**C**) The fluorescence intensity from panel (**B**) was quantitatively analyzed by flow cytometry. (**D**) Berberine and PDS, as positive control G4 stabilizers, were used to assess their inhibitory effects on PRV-GFP replication in PK-15 cells, as determined by flow cytometry. (**E**) PK-15 and Vero cells were infected with PRV-HN1201 (MOI = 0.1) and treated with varying concentrations of EPI. After 24 h, cellular DNA was extracted, and viral DNA levels were quantified by qPCR. (**F**) PK-15 cells were pre-treated with different concentrations of EPI and then infected with PRV-HN1201 at an MOI of 0.1 or 1. After 24 h of incubation with EPI, viral titers were determined using the TCID_50_ method. (**G**) PK-15 and Vero cells were treated as described in panel (**E**), and PRV gB protein expression was assessed by Western blot analysis. DMSO-treated samples were used as the negative control. All experiments were performed in triplicate. Data were analyzed by One-way ANOVA with Dunnett’s multiple comparisons test, * *p* < 0.05, ** *p* < 0.01, ***p < 0.001, ns: not significant.

To determine whether EPI affects PRV proliferation in live cells, we first evaluated the cytotoxicity of EPI on both PK-15 and Vero cells using the CCK-8 assay. The results showed that EPI concentrations below 40 µM did not significantly affect cell viability. However, at 80 µM, cell viability decreased to approximately 50% (Figure 6A). Based on these findings, we limited the maximum EPI concentration to 40 µM for subsequent experiments.

We then assessed the impact of EPI on PRV proliferation using the PRV-GFP strain. After infecting PK-15 and Vero cells with PRV-GFP, fluorescence microscopy revealed a substantial decrease in GFP intensity when various concentrations of EPI were added to the culture (Figure 6B). Flow cytometry further quantified this observation, confirming that EPI effectively inhibited PRV proliferation within the cells(Figure 6C).

As positive controls, the G4 stabilizers berberine and PDS also significantly inhibited PRV-GFP proliferation in PK-15 cells (Figure 6D), further supporting the role of G4 stabilization in suppressing viral replication. Additionally, the PRV gene copy number decreased in a concentration-dependent manner as the EPI concentrations increased (Figure 6E), which is consistent with the inhibitory effect of EPI on viral replication. Moreover, the TCID_50_ values for both MOI 0.1 and MOI 1 were significantly reduced in the presence of EPI (Figure 6F). Western blot analysis further validated these results, showing a significant reduction in viral protein expression (Figure 6G), corroborating the inhibitory effect of EPI on PRV proliferation.

These findings strongly indicate that EPI is capable of inhibiting PRV proliferation in infected cells.

### 2.7. EPI Selectively Inhibits the Replication Phase of PRV in Infected Cells

**Figure 7:**
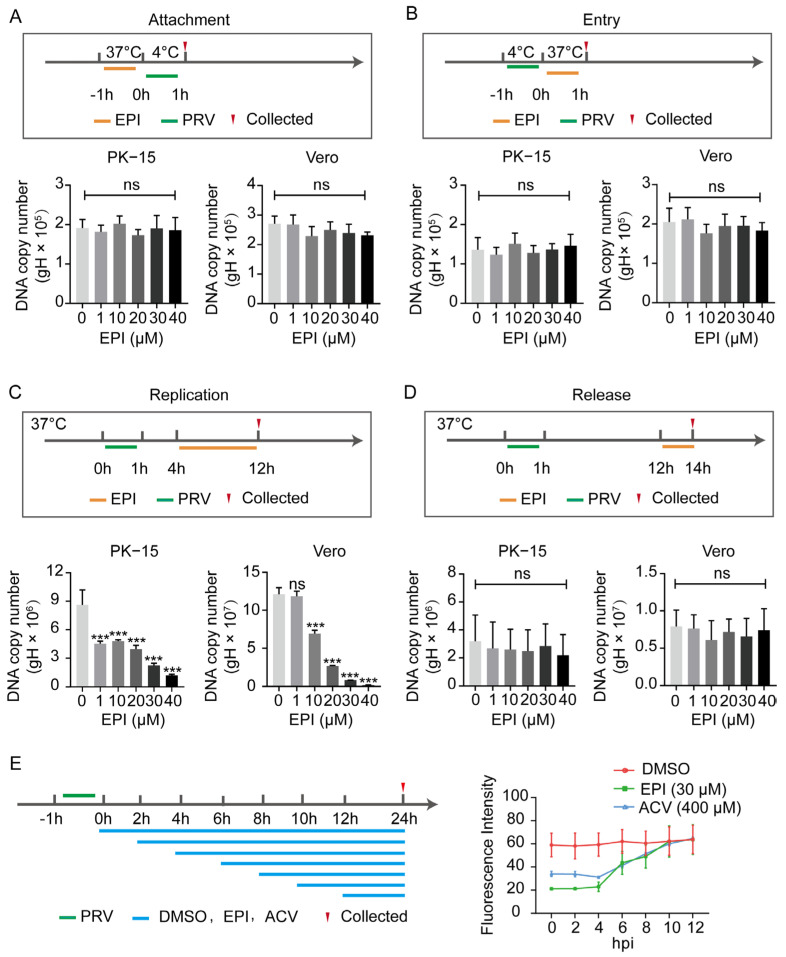
EPI impedes PRV replication in cells. (**A**–**D**) EPI inhibits PRV-HN1201 at different stages of the viral life cycle, including adsorption, entry, replication, and release, as assessed by qPCR. (**E**) Flow cytometry analysis of PRV-GFP infection in PK-15 cells treated with EPI or ACV. *** *p* < 0.001, ns: not significant.

Given that PRV infection in cells generally involves adsorption, invasion, replication, and release phases, we sought to determine at which stage EPI impacts PRV proliferation. As shown in Figure 7A–D, EPI primarily affects the replication phase of PRV within cells, with no significant impact on viral adsorption, invasion, or release. To further confirm this observation, we conducted a time-of-addition assay. EPI was added at different times post-infection at a concentration of 30 µM. Compared to the DMSO control, EPI significantly inhibited PRV gene copy number starting at 8 h post-infection, corresponding to the time when PRV replication occurs in cells. This effect was similar to that observed with the antiviral drug acyclovir (ACV) (Figure 7E), which is known to primarily target the replication process of PRV in cells.

These results collectively demonstrate that EPI specifically interferes with the replication phase of PRV’s life cycle, without affecting other stages of the viral infection process.

### 2.8. EPI Reduces Viral Load in PRV-Challenged Mice

**Figure 8:**
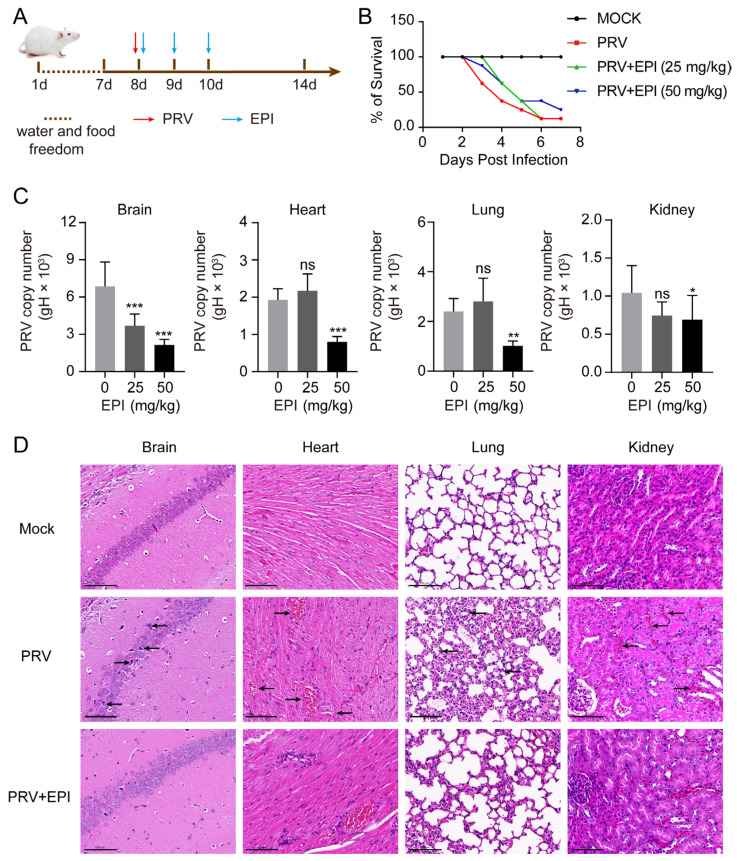
EPI inhibits PRV replication in mice. (**A**) Schematic diagram of the experimental procedure for treating PRV-infected mice with EPI. (**B**) Survival rates of mice in each group. Survival rates were calculated using the following formula: Survival rate = (Number of surviving mice/Total number of mice) × 100%. (**C**) Viral load in tissues from each group of mice. On day 3 post-PRV challenge, brain, heart, lung, and kidney tissues were collected, and viral DNA was extracted. The viral copy numbers in each tissue were determined by qPCR and compared to the DMSO-treated control group. (**D**) Histopathological examination of brain, heart, lung, and kidney tissues from mice; arrows indicate pathological lesions. Sections were stained with H&E, and the scale bar represents 100 μm. All experiments were performed in triplicate. Data were analyzed by one-way ANOVA with Dunnett’s multiple comparisons test, * *p* < 0.05, ** *p* < 0.01, *** *p* < 0.001, ns: not significant.

The above cell-based experiments confirmed that EPI effectively inhibits PRV replication. To further evaluate EPI’s inhibitory effects on PRV replication in vivo, a mouse challenge experiment was conducted, as outlined in Figure 8A. As shown in Figure 8B, mice began to exhibit significant neurological symptoms and mortality by 3 days post-infection (3 dpi), with survival rates dropping to 12.5% by 7 dpi compared to untreated mice. In the group treated with 25 mg/kg of EPI, although neurological symptoms and some mortality were observed by 3 dpi, fewer mice died compared to the PRV-challenged group. Treatment with 50 mg/kg EPI significantly improved survival rates, with a 12.5% increase in survival compared to the PRV-challenged group.

Next, we quantified PRV viral loads in the brain, heart, lung, and kidney tissues using qPCR. Compared to the DMSO control group, treatment with 50 mg/kg of EPI significantly reduced viral loads across all tissues, with the most pronounced effect observed in the brain, where 25 mg/kg EPI reduced viral load by approximately one-half (Figure 8C). Histopathological analysis further supported these findings. In PRV-challenged, untreated mice, significant proliferation of microglia was observed in brain tissues, cardiac tissues exhibited minor disruption and hemorrhaging of myocardial fibers, lung sections showed thickening of alveolar walls with inflammatory cell infiltration, and renal tissues displayed interstitial bleeding. However, in PRV-challenged mice treated with 50 mg/kg EPI, the proliferation of microglia in brain tissues was reduced, myocardial fiber hemorrhaging and interstitial bleeding in renal tissues were alleviated, and the thickening of alveolar walls in lung tissues was mitigated (Figure 8D).

These results demonstrate that EPI can effectively inhibit PRV replication in vivo.

## 3. Discussion

Pseudorabies virus, the causative agent of Aujeszky’s disease, has led to substantial economic losses in the swine industry. Moreover, PRV poses a zoonotic threat with potential cross-species transmission to humans, thereby representing a significant public health risk [61]. Therefore, there is an urgent need to comprehensively understand the virological characteristics of PRV and develop new antiviral strategies. One of the notable features of the PRV genome is its richness in guanine bases and the presence of multiple repeat sequences. In this study, we identified two guanine-rich repeat sequences, R1-PQS and R2-PQS, located on the sense and antisense strands of the genome, respectively. The distribution of these repeat regions in the genome is consistent with previous reports [49].

Using CD and fluorescence turn-on assays, we demonstrated that R1-PQS and R2-PQS can form hybrid quadruplex structures composed of four G-tetrads. This finding is distinct from previously reported G4 structures in the PRV genome. For example, we previously reported a parallel G4 structure with three G-tetrads near the replication origin Ori-L [39], and another study identified a two-G-tetrad RNA G4 structure at the 3′ end of the IE180 gene [62]. Interestingly, R1-PQS and R2-PQS are arranged in tandem repeats within the PRV genome, suggesting the potential formation of tandem G4 structures in vivo.

Our Taq polymerase stop assay revealed that the G4 structures formed by R1-PQS and R2-PQS on single-stranded templates hinder Taq polymerase extension, indicating that these G4s can impede the DNA replication process. Indeed, G4 structures in eukaryotic genomes have been shown to stall replication forks [63,64,65,66], suggesting that similar mechanisms may influence PRV replication. In addition, during DNA replication or transcription, the exposure of single-stranded regions creates favorable conditions for G4 formation. These G4 structures can act as physical barriers, affecting both replication and transcription. For example, G4s formed in gene promoter regions have been shown to impede RNA polymerase movement, thereby influencing transcription [67,68,69]. This suggests that the tandem G4 structures in the PRV genome may exert a stronger influence on DNA replication and transcription than single G4s, potentially explaining the biological function of the repeat regions located between genes in the PRV genome; the tandem G4s may serve to insulate against read-through transcription by providing steric hindrance. It is also important to note that other G4 motifs exist within the PRV genome, although most are present as single copies. Therefore, we cannot entirely rule out the possibility that EPI might affect PRV replication by targeting these other G4 structures in the viral genome as well.

G4 structures in cells are typically in a dynamic equilibrium between folded and unfolded states. Stabilization of these structures often requires the binding of specific G4 ligands, which can influence DNA replication, transcription, and translation. Consequently, some G4-stabilizing agents have demonstrated antiviral activity by binding to viral G4s [60,70]. In this study, we found that EPI, a natural compound, can bind to and stabilize the G4 structures formed by R1-PQS and R2-PQS, thereby inhibiting DNA replication (Figure 5). Further mouse experiments confirmed the inhibitory effect of EPI on PRV proliferation (Figure 8). This observation is consistent with previous reports showing that EPI can bind to and stabilize hybrid quadruplexes in telomeric DNA [59]. While G4 ligands typically interact with G4 structures through π-π stacking, the exact binding mode of EPI to the G4 structures formed by R1-PQS and R2-PQS requires further investigation.

## 4. Materials and Methods

### 4.1. Cells and Viruses

Porcine kidney (PK-15) cells (CCL-33, ATCC, Manassas, VA, USA) and African green monkey kidney (Vero) cells (CL-81, ATCC, Manassas, VA, USA) were cultured as monolayers at 37 °C in a humidified atmosphere containing 5% CO_2_. The cells were maintained in Dulbecco’s Modified Eagle’s Medium (DMEM; Cat# 10566-016, Gibco, Thermo Fisher Scientific, Waltham, MA, USA), supplemented with 10% fetal bovine serum (Cat# 10099141C, Gibco, Thermo Fisher Scientific, Waltham, MA, USA), 100 units/mL penicillin, and 100 µg/mL streptomycin sulfate (Cat# B540732, Sangon, Shanghai, China). The PRV-GFP recombinant PRV strain, derived from the Hubei strain of PRV with the thymidine kinase (TK) gene replaced by the green fluorescent protein (GFP) expression cassette from the pEGFP-N1 plasmid, was kindly provided by Dr. Hanzhong Wang from the Wuhan Institute of Virology, Chinese Academy of Sciences. The PRV-HN1201 strain was generously gifted by Professor KegongTian from Henan Agricultural University, China.

### 4.2. Materials and Oligonucleotides

TRIzol reagent (Cat# D9108B) and SYBR Premix Ex Taq (Cat# RR420A) were purchased from Takara Biotechnology Co., Ltd. (Dalian, China). The Cell Counting Kit-8 (CCK-8, Cat# ZP328, DingGuo, Beijing, China) was obtained from Zoman Biotechnology Co., Ltd. (Beijing, China). The TIANamp Virus DNA/RNA Kit (Cat# DP315) was sourced from Tiangen Biotech Co., Ltd. (Beijing, China). The animal tissue genomic DNA mini extraction kit was procured from Lifen Biotechnology Co., Ltd. (Shanghai, China). Thioflavin T (ThT, 98%) and N-Methyl Mesoporphyrin IX (NMM) were acquired from Bailinwei Technology Co., Ltd. (Beijing, China). EPI, Berberine, and Acyclovir (ACV) were purchased from Aladdin Biochemical Technology Co., Ltd. (Shanghai, China), BG4 antibodies were purchased from Sigma-Aldrich (MilliporeSigma, St. Louis, MO, USA), Flag antibodies were purchased from Proteintech Group, Inc. (Wuhan, China), DAPI were purchased from Servicebio, Inc. (Beijing, China), PDS was purchased from ChemeGen Corp. (Los Angeles, CA, USA). A primary antibody specific to PRV-gB was generated in-house. The sequences of the primers and other oligonucleotides used in this study are listed in Table S2.

### 4.3. Cell Viability Analysis

PK-15 and Vero cells were seeded at a density of 1 × 10^4^ cells per well in 96-well culture plates. When cells reached approximately 70% confluence, the growth medium was replaced with DMEM/10% FBS containing different concentrations of EPI. The cells were incubated for 24, 36, and 48 h. At each time point, 10 µL of CCK-8 reagent (Zoman Bio, Beijing, China) was added to each well, followed by a 3 h incubation at 37 °C. Cell viability was then assessed by measuring absorbance at 450 nm using a Varioskan Flash multifunctional microplate reader (Thermo Fisher Scientific, Waltham, MA, USA).

### 4.4. Flow Cytometry Assay

PK-15 and Vero cells were seeded at a density of 2 × 10^5^ cells per well in 12-well culture plates. Upon reaching 60% confluence, EPI, berberine, and PDS were added at various concentrations as per the experimental design. The cells were infected with PRV-GFP at a multiplicity of infection (MOI) of 0.1. Twenty-four hours post-infection, cells were detached using Trypsin-EDTA (Gibco, Waltham, MA, USA; purchased from Shanghai, China), collected by centrifugation, and resuspended in phosphate-buffered saline (PBS). The percentage of GFP-positive cells was measured using a CytoFLEX flow cytometer (Beckman Coulter, Brea, CA, USA). Data were acquired and analyzed using CytExpert software (version 2.4, Beckman Coulter, Brea, CA, USA).

### 4.5. Viral Titration

Vero cells were seeded into 96-well plates at a density of 1 × 10^4^ cells per well and cultured overnight. The cells were then inoculated with 10-fold serially diluted PRV and incubated for 1 h to allow viral attachment. After the incubation, the cells were washed with PBS to remove unbound virus, and fresh culture medium was added. The cells were cultured for 3 to 5 days, with daily observation for the presence of cytopathic effects (CPE). The TCID_50_ values were calculated using the Reed–Muench method.

### 4.6. Western Blot Assay

PK-15 and Vero cells were seeded at a density of 2 × 10^6^ cells per 60 mm dish. Once cells reached 60% confluence, they were infected with PRV-HN1201 at a multiplicity of infection (MOI) of 0.1 for 1 h. After infection, cells were washed with pre-chilled PBS and lysed in RIPA buffer containing 1 mM PMSF for 10 min. The lysate was centrifuged at 13,000× *g* for 10 min at 4 °C, and the protein supernatant was collected and quantified using a BCA Protein Assay Kit (Shanghai Bioscience, Shanghai, China). The protein samples were subjected to 10% SDS-PAGE and then transferred onto a PVDF membrane (IPVH00010; Millipore, Burlington, MA, USA).The membrane was blocked with PBS containing 5% non-fat dry milk and 0.1% Tween 20 at 25 °C for 1 h. It was then incubated with the primary antibody overnight at 4 °C, followed by incubation with the secondary antibody at 25 °C for 1 h. Chemiluminescent detection was performed using the SuperSignal West Femto substrate (Thermo Fisher Scientific, Waltham, MA, USA), and signals were captured using a chemiluminescence imaging system (GE Healthcare, Boston, MA, USA).

### 4.7. DNA Extraction and Quantitative Polymerase Chain Reaction

PK-15 and Vero cells were seeded at a density of 2 × 10^5^ cells per well in 12-well plates. After a 1 h infection with PRV-HN1201 at a multiplicity of infection (MOI) of 0.1, cells were treated with varying concentrations of EPI and collected at designated time points. DNA was extracted using the TIANamp Virus DNA/RNA Kit following the manufacturer’s instructions. Real-time quantitative PCR was performed using SYBR Premix Ex Taq to quantify PRV genome copies. The PRV gH gene (187 bp) was cloned into the pGEM-T Easy vector (Promega Corporation, Madison, WI, USA) to create the recombinant plasmid pGEM-gH, which served as a standard for generating a standard curve to accurately quantify viral DNA in the qPCR assay.

### 4.8. Circular Dichroism Spectroscopy

Wild-type and mutant single-stranded DNA were diluted to a final concentration of 10 µM in 25 mM Tris-HCl buffer (pH 7.4) containing 50 mM KCl. The samples were heated to 95 °C for 5 min, then slowly cooled to room temperature overnight, and subsequently incubated with EPI at 25 °C for 2 h. CD spectra were recorded from 220 to 320 nm at 25 °C using a Chirascan-plus circular dichroism spectrometer (Applied Photophysics, Leatherhead, UK), with a 0.5 mm light path and 1 nm bandwidth. Buffer was used as a baseline for spectral correction.

For the melting assay, annealed oligonucleotides incubated with EPI were heated from 20 °C to 98 °C at a rate of 1 °C/min, with CD ellipticity recorded at the peak wavelength. The melting temperature (Tm) was determined by fitting a sigmoidal curve to the data using the Boltzmann function in Prism 8.0 software (GraphPad Software, San Diego, CA, USA).

### 4.9. Fluorescence Turn-On Assay

This experimental procedure was adapted from [29] with minor modifications. Oligonucleotides were diluted to 0.3 µM in a buffer containing 25 mM Tris-HCl (pH 7.4) and 50 mM KCl. The samples were heated at 95 °C for 5 min, then cooled to 25 °C at a rate of 0.01 °C/s. After cooling, the samples were incubated with 0.6 µM Thioflavin T (ThT) or 0.6 µM N-Methyl Mesoporphyrin IX (NMM) at 25 °C for 10 min. Fluorescence measurements were taken using a multifunctional microplate reader. For ThT, the excitation wavelength was set to 425 nm, and fluorescence signals were recorded from 450 to 700 nm at 2 nm intervals. For NMM, the excitation wavelength was set to 392 nm, with signals recorded from 500 to 700 nm at 2 nm intervals. Additionally, with EPI fixed at 0.2 μM, the oligonucleotide concentrations were gradually increased, and fluorescence was recorded from 400 to 700 nm using a fluorescence spectrophotometer.

### 4.10. Taq Polymerase Stop Assay

This experimental procedure was adapted from [29] with minor modifications. Single-stranded templates (1 µM, wild-type or mutant) were incubated with 1.2 µM of 5′-FAM-labeled primers and heated at 95 °C for 5 min in a buffer containing 50 mM KCl and 25 mM Tris-HCl. The samples were then slowly cooled to 25 °C at a rate of 0.01 °C/s. Following cooling, varying concentrations of EPI, berberineor PDS were added. Next, 2.5 U of Taq DNA polymerase (Takara Bio Inc., Dalian, China) was introduced, and primer extension was carried out at 60 °C for 30 min. The reaction was terminated by ethanol precipitation, and the extension products were separated on a 20% polyacrylamide gel containing 7 M urea. Gels were analyzed using an Amersham Imager 600 (GE Healthcare, Boston, MA, USA), and the ratio of prematurely terminated products was calculated using Prism 8.0 (GraphPad Software, San Diego, CA, USA).

### 4.11. Immunofluorescence Analysis

PK-15 and 293T cells (1 × 10^5^) were seeded in 24-well culture plates containing coverslips. For 293T cells, when the cell density reached 60%, they were transfected with 1 μg of FAM-labeled oligonucleotide DNA using TurboFect (purchased from Thermo Fisher Scientific, Waltham, MA, USA) and then incubated for 24 h. For PK-15 cells, when they reached approximately 60% confluence, they were infected with PRV-HN1201 (MOI = 1). One hour post-infection, the medium was replaced with fresh maintenance medium and then collected at the designated time points (0, 2, 4, 6, 8, 10, and 12 h). Subsequently, cells were fixed with 4% paraformaldehyde (prepared in PBS) for 30 min, followed by permeabilization with 0.1% Triton X-100 (prepared in PBS). After permeabilization, cells were blocked with 10% fetal bovine serum (FBS) in PBS for 1 h. Then, BG4 antibody was used to incubate cells overnight at 4 °C. On the following day, for cells transfected with oligonucleotides, Flag antibody (diluted in PBS with 10% FBS) was used as the primary antibody, incubating at room temperature for 1 h. Subsequently, the secondary antibody, Alexa Fluor 568-conjugated goat anti-rabbit IgG (purchased from Thermo Fisher Scientific, Waltham, MA, USA), was used at room temperature for 1 h.

For PK-15 cells infected with PRV, Flag, and UL42 antibodies (both diluted in PBS with 10% FBS) were used as the primary antibodies and incubated at room temperature for one hour. As secondary antibodies, Alexa Fluor 568-conjugated goat anti-rabbit IgG and Alexa Fluor 488-conjugated goat anti-mouse IgG from Thermo Fisher Scientific (Waltham, MA, USA) were used and incubated at room temperature for 1 h.

Finally, DAPI was applied for 12 min to stain the cell nuclei. Immunofluorescence images were acquired using a Zeiss LSM 800 confocal microscope (Carl Zeiss, Jena, Germany).

### 4.12. Experiments in Mice

Forty-four female C57BL/6J mice (25 ± 2 g) (License number: SCXK(Liao)2020-0001) were obtained from Liaoning Changsheng Biotechnology Co., Ltd. (Liaoning, China) and housed in a specific pathogen-free facility. The mice were randomly divided into four groups: a normal control group, a PRV-challenged group, and two PRV-challenged groups treated with EPI. Each group contained 11 mice. Except for the normal control group, all mice were intranasally inoculated with 5000 TCID_50_ of PRV-HN1201. One hour post-inoculation, the PRV-challenged groups were treated with intraperitoneal injections of EPI at doses of 25 mg/kg or 50 mg/kg, administered once daily for three consecutive days. The normal control group and the PRV-challenged control group received an equivalent volume of DMSO. On the third day post-virus inoculation, three mice from each group were randomly selected for necropsy to evaluate viral load and tissue pathology. The remaining mice were monitored throughout the experimental period to assess survival rates. To quantify viral load, DNA was extracted from brain, heart, lung, and kidney tissues and analyzed using quantitative PCR (qPCR). Additionally, tissue samples were fixed in 4% paraformaldehyde, embedded, and sectioned for histopathological examination using hematoxylin and eosin (H&E) staining.

### 4.13. Statistical Analysis

Data were obtained from at least three independent experiments for quantitative analyses and are expressed as means ± standard errors of the means. All the statistical analyses were performed with one-way analysis of variance (ANOVA) or *t*-test using Prism 8.0 (GraphPad Software, San Diego, CA, USA). Significant differences relative to the corresponding controls were accepted at * *p* < 0.05, ** *p* < 0.01, and *** *p* < 0.001, and ns indicates no significant difference.

## 5. Conclusions

In summary, this study identified two tandem G4 structures in the PRV genome, advancing our understanding of the genomic features of PRV. Furthermore, we demonstrated that EPI inhibits PRV replication by binding to these G4 structures, suggesting that EPI is a promising candidate for antiviral drug development.

## Data Availability

The data presented in this study are available in both the article and Supplementary Materials.

## Supplementary Materials

The following supporting information can be downloaded at https://www.mdpi.com/article/10.3390/ijms26083764/s1.
