# Supplementary material for: Tandemly Repeated G-Quadruplex Structures in the Pseudorabies Virus Genome: Implications for Epiberberine-Based Antiviral Therapy
Source: Int J Mol Sci. 2025 Apr 16;26(8):3764. doi: 10.3390/ijms26083764 (PMC12028228; doi:10.3390/ijms26083764)
Supplement: Supplementary file 1 [file ijms-26-03764-s001.zip › Table S1.pdf]

Table S1: Sequences of the repeated G4s in the PRV genome.

HN1201<br>R1-PQS<br>(+ strand)	16381-ACAAGGCGTGTGTGTCGTGGCGGTCGTGGTCGCGGG<br>ACGGGAGGGCGGCGGGGAGAGGGGAGACGAGAGGGGAGA<br>GGGGAGACGAGAGGGGAGAGGGGAGACGAGAGGGGAGAG<br>GGGAGACGAGAGGGGAGAGGGGAGACGAGAGGGGAGAGG<br>GGAGACGAGAGGGGAGAGGGGAGACGAGAGGGGAGAGGG<br>GAGACGAGAGGGGAGAGGGACGAGGCGGACGCGAGACGT<br>GCGATCAACGGCATCTTTATTTTTTTCCA-16640
hSD-1/2019<br>R1-PQS<br>(+ strand)	15601-CGTGTGTGTCGTGGCGGTCGTGGTCGCGGGACGGGA<br>GGGCGGCGGGGAGAGGGGAGACGAGAGGGGAGAGGGGAG<br>ACGAGAGGGGAGAGGGGAGACGAGAGGGGAGAGGGGAGA<br>CGAGAGGGGAGAGGGGAGACGAGAGGGGAGAGGGGAGAC<br>GAGAGGGGAGAGGGGAGACGAGAGGGGAGAGGGGAGACG<br>AGAGGGGAGAGGGGAGACGAGAGGGGAGAGGGACGAGGC<br>GGACGCGAGACGTGCGATCAACGGCATC-15860
JS-2012<br>R1-PQS<br>(+ strand)	16511-GGTCGCGGGACGGGAGGGCGGCGGGGAGAGGGGAG<br>ACGAGAGGGGAGAGGGGAGACGAGAGGGGAGAGGGGAGA<br>CGAGAGGGGAGAGGGGAGACGAGAGGGGAGAGGGGAGAC<br>GAGAGGGGAGAGGGGAGACGAGAGGGGAGAGGGGAGACG<br>AGAGGGGAGAGGGGAGACGAGAGGGGAGAGGGGAGACGA<br>GAGGGGAGAGGGACGAGGCGGACGCGAGACGTGCGATCAA<br>CGGCATCTTTAT-16753
HN1201<br>R2-PQS<br>(- strand)	32568-CAAGTCTTTGGGTCCCACAACCCCCAAGTTTTGGGGT<br>CTCGGGTCCGAGTCCCCCGAGTCCCCCGAGTCCCCCGAGTC<br>CCCCGAGTCCCCCGAGTCCCCCGAGTCCCCGAGTCCCCCGA<br>GTCCCCGAGTCCCCCGAGTCCCCGAGTCCCCCGAGTCCCCG<br>AGTCCCCCGAGTCCCCCGAGTCCCCCGAGTCCCCGAGTCCC<br>CGAGTCCCCCGAGTCCCCCGAGTCCCCCGAGTCCCCCGAGT<br>CCCCCGAGTCCCCCGAGTCCCCCGAGTCCCCCGAGTCCCCG<br>AGTCCCCCGAACACACCACCGCAGAGACAAACAGGTTGGG-<br>32890
hSD-1/2019<br>R2-PQS<br>(- strand)	31801-CAAGTCTTTGGGTCCCACAACCCCCAAGTTTTGGGGT<br>CTCGGGTCCGAGTCCCCCGAGTCCCCCGAGTCCCCCGAGTC<br>CCCCGAGTCCCCCGAGTCCCCCGAGTCCCCCGAGTCCCCCG<br>AGTCCCCCGAGTCCCCCGAGTCCCCCGAGTCCCCCGAGTCC<br>CCCGAGTCCCCCGAGTCCCCCGAGTCCCCGAGTCCCCCGAG<br>TCCCCCGAGTCCCCCGAGTCCCCCGAGTCCCCCGAGTCCCC<br>GAGTCCCCCGAACACACCACCGCAGAGACAAACAGGTTGG<br>GTAATAAACAATTTATTAACCAAGAATC-32110
JS-2012<br>R2-PQS	32722-GTCCCACAACCCCCAAGTTTTGGGGTCTCGGGTCCGA<br>GTCCCCCGAGTCCCCCGAGTCCCCCGAGTCCCCCGAGTCCC<br>CCGAGTCCCCCGAGTCCCCCGAGTCCCCCGAGTCCCCCGAG<br>TCCCCCGAGTCCCCCGAGTCCCCCGAGTCCCCCGAGTCCCC

(- strand)	GAGTCCCCCGAGTCCCCCGAGTCCCCCGAGTCCCCCGAGTC<br>CCCCGAGTCCCCCGAACACACCACCGCAGAGACAAACAGG<br>TTGGGTAATAAACAATTTATTA-32984
