# Supplementary material for: Tandemly Repeated G-Quadruplex Structures in the Pseudorabies Virus Genome: Implications for Epiberberine-Based Antiviral Therapy
Source: Int J Mol Sci. 2025 Apr 16;26(8):3764. doi: 10.3390/ijms26083764 (PMC12028228; doi:10.3390/ijms26083764)
Supplement: Supplementary file 1 [file ijms-26-03764-s001.zip › Table S2.pdf]

Table S2. Oligonucleotides and primer sequences

Primer	Sequence (5′ to 3′) *
**qPCR**	
R1-PQS-F	CCACGGACTCACCGTTGTATTG
R1-PQS-R	GGCCACGCGCCGCCGGCACTA
R2-PQS-F	CAACCCCCAAGTTTTGGGGTCT
R2-PQS-R	TGTCTCTGCGGTGGTGTGTT
PRV-gH-F	CTCGCCATCGTCAGCAA
PRV-gH-R	GCTGCTCCTCCATGTCCTT
**CD and CD melting assay**	
R1-PQS-WT	**GGGG**AGA**GGGG**AGACGAGA**GGGG**AGA**GGGG**
R1-PQS-Mut	G<u>A</u>G<u>A</u>AGAG<u>AA</u>GAGACGAGAG<u>AA</u>GAGAG<u>A</u>GG
R2-PQS-WT	**GGGG**ACTC**GGGGG**ACTC**GGGGG**ACTC**GGGG**
R2-PQS-Mut	GG<u>A</u>GACTC<u>A</u>G<u>A</u>GGACTC<u>A</u>G<u>AA</u>GACTC<u>A</u>G<u>A</u>G
Bcl-2	**GGG**CGC**GGG**AGGAA**GGGGG**C**GGG**
TA[Q]	TAGGGTTAGGGTTAGGGTTAGGG
**Taq polymerase termination assay**	
R1-WT-Template	TTTTTGGGGAGAGGGGAGACGAGAGGGGAGAGGGGTTTTTCGCA*CTGAGCGAAGATACGGAGCCACGCCA*
R1-Mut-Template	TTTTTGAGAAGAGAAGAGACGAGAGAAGAGAGAGGTTTTTCGCA*CTGAGCGAAGATACGGAGCCACGCCA*
R2-WT-Template	TTTTTGGGGACTCGGGGGACTCGGGGGACTCGGGGTTTTTCGCA*CTGAGCGAAGATACGGAGCCACGCCA*
R2-Mut-Template	TTTTTGGAGACTCAGAGGACTCAGAAGACTCAGAGTTTTTCGCA*CTGAGCGAAGATACGGAGCCACGCCA*
hTel-Template	TTTTTAGGGTTAGGGTTAGGGTTAGGGTTTTTCGCA*CTGAGCGAAGATACGGAGCCACGCCA*
Primer	FAM-TGGCGTGGCTCCGTATCTTCGCTCAG

* Gs that participate in G4 folding are shown in bold, and the mutated bases are underlined. For the Taq polymerase termination experiment, the complementary primer region is indicated by italics.
